# Anti-Inflammatory Effect of *Aniba rosaeodora* Essential Oil-Loaded Chitosan Membrane

**DOI:** 10.1021/acsomega.5c06864

**Published:** 2026-02-18

**Authors:** Bruna Michele A. de B. Buriti, Caio Augusto de Almeida Canelas, Venâncio A. Amaral, Laine Celestino Pinto, Renata C. Silva, William N. Setzer, Tais Gratieri, Pablo Luis B. Figueiredo, Marcele F. Passos, Joyce Kelly R. da Silva

**Affiliations:** † Instituto de Ciências Exatas e Naturais, Programa de Pós-Graduação em Química, 37871 Universidade Federal do Pará, Belém, Pará 66075-110, Brazil; ‡ Programa de Pós-Graduação em Ciências Farmacêuticas, Universidade Federal do Pará, Belém, Pará 66079-420, Brazil; § Laboratório de Alimentos, Medicamentos e Cosméticos (LTMAC), Universidade de Brasília, Brasília, Distrito Federal 70910-900, Brasil; ∥ Laboratório de Neuropatologia Experimental, Hospital Universitário João de Barros Barreto, Universidade Federal do Pará, Belém, Pará 66073-000, Brazil; ⊥ Programa de Pós-Graduação em Biologia Parasitária na Amazônia, Universidade Estadual do Pará, Belém, Pará 66095-662, Brazil; # Aromatic Plant Research Center, 230 N 1200E, Suite 100, Lehi, Utah 84043, United States; ∇ Programa de Pós-Graduação em Biotecnologia, Universidade Federal do Pará, Belém, Pará 66075-110, Brazil

## Abstract

Bioactive chitosan (CH) membranes containing 0.5, 2.5, and 5.0% w/w of *Aniba rosaeodora* essential oil (ArEO), rich in linalool (82.9%) and cis-linalool oxide (2.9%) were developed using the casting method. Fourier transform infrared spectroscopy (FTIR) results demonstrated that CH chains interacted with ArEO, leading to increased thermal stability and preventing isolated ArEO degradation. ArEO concentrations did not influence membrane morphology. Incorporating ArEO into the formulated membranes did not significantly alter their Young’s modulus (41.84–48.00 MPa), which remains within the skin’s modulus of elasticity range (0.02 to 57 MPa). They also exhibited mucoadhesion for tissue adhesion, with no significant difference with adding ArEO (0.135–0.161 N). Additionally, ArEO incorporated into the developed wound dressings significantly reduced nitrite levels in the in vitro inflammation model. Tumor necrosis factor-alpha (TNF-α) levels in lipopolysaccharide (LPS)-stimulated macrophages were significantly reduced at all ArEO concentrations in the wound dressings. The developed membranes may represent a promising alternative for wound dressings with anti-inflammatory properties.

## Graphical abstract

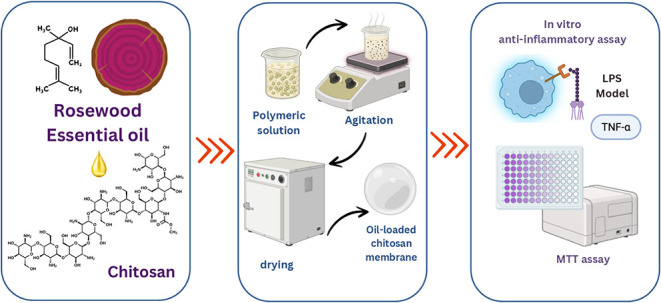

## 1 Introduction

Tissue engineering is a multidisciplinary area that integrates materials science with biological and engineering principles to develop systems that support tissue regeneration.^1^ In recent years, the growing demand for effective therapeutic solutions to enhance the quality of life has stimulated the design of innovative and intelligent biomaterials aimed at promoting tissue and organ regeneration.^2,3^

The healing process involves four distinct phases: hemostasis, inflammation, proliferation, and remodeling. Initially, coagulation factors activate, forming a platelet plug, minimizing blood loss. Inflammation releases proteolytic enzymes and pro-inflammatory cytokines. The proliferation phase, angiogenesis occurs, leading to scar formation. Finally, during the remodeling phase, newly formed capillaries regress, and most macrophages and fibroblasts undergo apoptosis.^4^ In parallel, the use of an appropriate sterile dressing is essential to restore the regenerative properties of the skin and to act as a protective barrier against the external environment, simulating the natural epithelial function.^5,6^

A perfect wound dressing should keep the area moist, facilitate gas exchange, absorb fluids, support cell growth, and prevent bacterial infection.^7^ Membrane dressings are particularly advantageous due to their ability to absorb fluids without leaks, reduce change frequency, protect against microorganisms, and maintain flexibility and transparency, which enhances healing.^8^ Polysaccharide-based compounds like chitosan, alginates, and cellulose derivatives are popular for membrane preparation due to their biodegradability, biocompatibility, a strong ability to create delivery systems for bioactive substances, and cost-effectiveness.^3,9^ Chitosan specifically offers benefits such as promoting blood clotting and regulating inflammatory cell activity. It also possesses excellent antimicrobial, antioxidant, and hemostatic properties, making it a promising material for wound healing.^10–13^

There is a growing interest in utilizing natural healing composed of medicinal plant derivatives to create innovative topical treatments for skin wounds.^14^ Essential oils (EOs) are volatile aromatic substances known for their phenylpropanoid and terpenoid components.^15^ These oils possess antioxidant and antibacterial properties, in addition to exhibiting antiviral, insecticidal, analgesic, and anti-inflammatory effects.^16^ EOs can promote faster wound healing, contribute to collagen formation, and stimulate fibroblast growth during the healing process.^17,18^

The essential oil derived from *Aniba rosaeodora* Ducke (rosewood), a species native to the Amazon, contains a high proportion of linalool (about 85–90%). Its recurrent use in aromatherapeutic applications and in treatments targeting skin regeneration and regulation of immune and nervous system functions underscores its importance as an ethnopharmacologically valuable natural product.^19,20^ Several clinical studies have shown that *Aniba rosaeodora* EO, high in linalool, exhibits antidepressant activity and has potential anesthetic properties.^21–23^ Linalool, a monoterpene, demonstrates various biological activities, including antibacterial, anti-inflammatory, antihyperalgesic, antinociceptive, anticancer, anxiolytic, and antiplasmodial effects.^24,25^

Incorporating essential oils (EOs) into polymer membranes aims to overcome limitations like volatility and low stability issues while enhancing antibacterial, barrier, and mechanical properties.^26,27^ Recent bioactive membranes have demonstrated excellent healing capabilities due to their tissue adhesive qualities and adaptability to wound movement.^2,3^ Notable developments include tea tree oil-loaded chitosan hydrogels, which demonstrated antibacterial activity and significantly accelerated wound healing,^28^ while *Perovskia abrotanoides* EO-loaded chitosan gel may be useful for promoting wound healing due to its ability to prevent infections and speed up the recovery process.^29^ Additionally, chitosan/poly­(vinyl alcohol) films with sweet fennel oil exhibit improved elasticity, hydrophilicity, water absorption, and porosity, benefiting wound healing.^30^

Supplementing a fundamental membrane system with a bioactive component is a novel approach. Challenges like the risk of infection, immune responses, high expenses, and limited supply can be addressed by utilizing naturally derived polymers. Therefore, the objective of the present work was to evaluate the influence of the incorporation of *Aniba rosaeodora* EO, a unique Amazonian natural resource rich in linalool, in chitosan-based membranes and to compare the chemical, thermal, morphological, mechanical, and anti-inflammatory properties for application in the biomedical sector. Although several studies have investigated biopolymers combined with natural compounds as anti-inflammatory agents, no reports exist on chitosan membranes incorporating specific Amazonian bioactive compounds for potential application in the inflammatory phase of wound healing. This combination is expected to confer not only the well-established biocompatibility and film-forming properties of chitosan, but also additional and specific anti-inflammatory activity derived from Amazon rosewood oil. Thus, this work provides an original contribution by proposing a novel bioactive membrane that addresses a critical stage of wound healing, which remains underexplored in the literature, while also offering a sustainable use of Amazonian biodiversity for biomedical applications.

## 2 Materials and Methods

### 2.1 Plant Material and Essential Oil Extraction

Leaves of a wild specimen of *Aniba rosaeodora* Ducke were collected in the Campus of Universidade Federal Rural da Amazônia (UFRA). The voucher was registered at the Herbarium of Museu Paraense Emílio Goeldi in Belém, Brazil (MFS 10553). The activity of access to the Genetic Heritage was registered in SisGen under registration number AF50EA1. The leaves were dried at room temperature and ground and then submitted to EO extraction using a Clevenger-type apparatus. The *Aniba rosaeodora* essential oil (ArEO) was analyzed on a GC-MS. Linalool (82.9%) and *cis*-linalool oxide (2.9%) were the major constituents.^20^

### 2.2 Membrane Preparation

Commercial chitosan (CH, with approximately 77.28% deacetylation) was provided by Exodo Científica (São Paulo, Brazil). The membranes were obtained using the casting method. To prepare for the CH solution, 1% w/v chitosan was dissolved in acetic acid. The mixture was continuously stirred using a magnetic stirrer for 24 h at room temperature, approximately 25 °C. The stirring was maintained at 500 rpm to ensure complete dissolution and homogenization of the chitosan.

After the complete dissolution of CH, the essential oil of *Aniba rosaeodora* was gradually added. The addition was made continuously while the mixture was kept at 500 rpm to ensure uniform oil distribution in the chitosan solution. The mixture was homogenized for 30 min at room temperature. The final concentrations of ArEO were 0.5% (CH/ArEO 0.5%), 2.5% (CH/ArEO 2.5%), and 5.0% (CH/ArEO 5.0%). The viscosity of the samples was evaluated using a rotational viscometer, as detailed in the Supporting Information.

The solution was placed in flat molds and then subjected to drying in an oven with circulating air at a temperature of 40 °C for 24 h. This process was carried out to guarantee that the solvent fully evaporated and the material solidified completely. The temperature was monitored throughout the drying process to avoid variations that could affect the membranes’ final structure. After drying, the membranes were removed and stored in a desiccator to preserve their properties before testing. The thickness of the membranes was 0.24 ± 0.016 mm. Macroscopic image of synthesized membrane can be viewed in the Supporting Information (Figure S1).

### 2.3 Characterization of Membranes

#### 2.3.1 Scanning Electron Microscope (SEM)

The morphological analysis of the polymeric membranes was performed using scanning electron microscopy (SEM JEOL, JSM-7000 IF, USA) using a secondary electron (SE) detector. Membranes with an area of 1 × 1 mm fixed in a sample holder (stub) were used for the analysis. The samples were then metalized with gold and analyzed at 10 and 100 μm magnifications.

#### 2.3.2 Thermogravimetric Analysis (TGA)

Thermogravimetric analyses were performed using a DTG-60H analyzer (Shimadzu, Kyoto, Japan) equipped with platinum crucibles under a nitrogen atmosphere at a constant flow rate of 50 mL·min^–1^. Samples weighing approximately 3–5 mg were heated from 25 to 500 °C at a rate of 10 °C·min^–1^. Measurements were conducted for both ArEO and membrane samples. The first derivative thermogravimetric (DTG) curves were obtained from the TGA data and analyzed using TA-60 software (Shimadzu, Kyoto, Japan). Graphical representations were prepared with OriginPro 2018 (OriginLab Corp., Northampton, MA, USA).

#### 2.3.3 Fourier Transform Infrared Spectroscopy (FTIR)

FTIR spectra were acquired using a Vertex 70v spectrometer (Bruker, Madison, WI, USA) equipped with an attenuated total reflectance (ATR) accessory operating in transmittance mode. Spectra were collected over the range of 4000–400 cm^–1^ with a resolution of 2 cm^–1^, averaging 60 scans for each sample.

#### 2.3.4 Mechanical Properties

The mechanical properties of the membranes were evaluated using a texture profile analyzer (TA.XT Plus, Stable Micro Systems, Surrey, UK) equipped with a 5 kg load cell, following the ASTM D882–02 standard.^31^ Tensile tests were performed to determine the breaking strength, elongation, breaking strain, and deformation (*n* = 4). Membrane specimens with an area of 5 cm^2^ were securely mounted in the grips, with an initial gauge length of 30 mm. The test speed was set to 2 mm·s^–1^, and an initial force of 0.049 N was applied (Tensile Grips, ref: TRT1/TG). Samples were elongated until failure, and stress was calculated as the ratio of the applied force to the cross-sectional area. Young’s modulus was calculated by the relationship between the tension and deformation of the membranes after applying a tensile force. Elongation (Δ*l*) was obtained as the difference between the final (*l*_f_) and initial (*l*_0_) lengths, and the percentage deformation (ε) was calculated according to eq 1:

1 $$\varepsilon \left(\%\right)=\left(\Delta l/l_{0}\right)\times 100$$

Where Δ*l* represents the change in length, while *l*_0_ denotes the original length, with both measurements expressed in millimeters (mm).

To evaluate the mucoadhesive capacity of the membranes, an 8% (m/v) mucin solution in ultrapure water was prepared to prepare the mucin discs. The discs were prepared using 250 mg of mucin and 150 μL of the mucin solution, forming discs with a diameter of 9 mm. The analysis was conducted utilizing a texture profile analyzer (TA.XT Plus, Stable Micro Systems, Surrey, UK) in compression force mode (Adhesive Test). The membranes were fixed on an appropriate platform, and the mucin disc was fixed on the lower end of the analytical probe (SMS P/10). Then, the mucin disc was compressed onto the membrane surface from the apical to basal direction with an applied force of 0.2 N. The contact time between the disc and the membrane was maintained for 30 s, and the probe displacement rate was set to 0.1 mm·s^–1^. The detachment force was calculated from the force (N) × time (s) ratio required to separate the mucin disc from the membrane surface. All measurements were performed in quadruplicate.

### 2.4 Biological Tests

#### 2.4.1 Cell Viability Analysis

Cell viability was assessed using the MTT [3-(4,5-dimethylthiazol-2-yl)-2,5-diphenyltetrazolium bromide] assay, based on the reduction of the yellow tetrazolium salt to a purple formazan product.^32^ Membranes and essential oils were evaluated for cytotoxic activity against the RAW 264.7 murine macrophage cell line. Cells were cultured in Dulbecco’s Modified Eagle’s Medium (DMEM, Gibco) supplemented with 10% fetal bovine serum (Nova Biotecnologia, 10-bio500) and 1% penicillin–streptomycin solution (10,000 U·mL^–1^, Sigma-Aldrich, P4333). Cultures were maintained at 37 °C for 24 h in a humidified incubator containing 5% CO_2_.

After the incubation and cell adhesion period, cells were treated with the essential oil at concentrations of 250.0, 125.0, 62.5, 31.3, 15.6, and 7.8 μg·mL^–1^, previously diluted in dimethyl sulfoxide (DMSO), and distributed into 96-well plates (100 μL per well). DMSO (0.01%) was used as a negative control. Following 24 h of treatment, the plates were centrifuged at 1500 rpm for 15 min, and the supernatant was discarded. Subsequently, 100 μL of MTT solution prepared in serum-free medium (final concentration of 0.5 mg·mL^–1^) was added to each well, and the plates were incubated for 3 h under 5% CO_2_. The plates were then centrifuged at 3000 rpm for 10 min, and the supernatant was carefully removed. The resulting formazan crystals were dissolved by adding 100 μL of DMSO and shaking for 10 min. Absorbance was measured at 570 nm using a microplate reader (Polaris-Celer, Celer Biotecnologia, Belo Horizonte, Brazil).

#### 2.4.2 In Vitro Anti-Inflammatory Activity

The concentrations of essential oil used for the anti-inflammatory assays were selected based on the MTT cell viability results, choosing three concentrations that did not affect macrophage viability. RAW 264.7 murine macrophages were cultured at a density of 1 × 10^5^ cells per well in 96-well plates and incubated for 24 h at 37 °C in a humidified atmosphere containing 5% CO_2_ to allow cell adhesion. After incubation, the cells were stimulated with lipopolysaccharide (LPS) from *Escherichia coli* O111:B4 (Sigma-Aldrich, L2630) at a concentration of 0.5 μg·mL^–1^ for 2 h. Subsequently, the cells were treated or left untreated (LPS control group) with the three selected essential oil concentrations and further incubated for 24 h under the same conditions. Dexamethasone (10 μM) was used as a positive control, while DMSO (0.01%) served as a negative control to confirm the effectiveness of the inflammation model. After treatment, the culture supernatants were collected for analysis. Nitrite levels were determined using the Griess reaction, and the remaining supernatants were stored at −80 °C for subsequent cytokine quantification.

##### 2.4.2.1 Nitrite Dosage

The detection and quantification of nitrite were carried out using a colorimetric method. This process involves the formation of an azo dye resulting from the reaction between nitrites in the sample and a Griess reagent, which is generally composed of sulfanilamide and *N*-(1-naphthyl)­ethylenediamine dihydrochloride (NED).^33^ For the assay, 50 μL aliquots of the culture supernatants were mixed with an equal volume of the modified Griess reagent (Sigma-Aldrich, G4410) and incubated in 96-well plates at room temperature for 10 min in the dark. Following this incubation, the absorbance of the samples was measured using a microplate reader (LMR-96, Loccus, Brazil) at a wavelength of 540 nm. A nitrite standard curve ranging from 0 to 100 μM was established to assess the nitrite concentration in the samples.

##### 2.4.2.2 Measurement of Tumor Necrosis Factor-Alpha (TNF-α) through ELISA

The concentration of tumor necrosis factor alpha (TNF-α) was determined using a commercial ELISA kit (Sigma-Aldrich, RAB1089) following the procedure recommended by the manufacturer. In brief, 100 μL of each culture supernatant was transferred to duplicate wells and incubated overnight at 4 °C. After the plates were washed four times, 100 μL of the prepared 1× detection antibody solution was added, and the plates were incubated for 1 h at room temperature. The washing step was repeated, and 100 μL of the streptavidin solution was added, followed by incubation for 45 min at room temperature. The colorimetric reaction was developed by adding the 3,3′,5,5′-tetramethylbenzidine (TMB) substrate and allowing it to react for 30 min. Absorbance was read at 450 nm using a microplate reader (Polaris-Celer, Celer Biotecnologia, Belo Horizonte, Brazil). TNF-α concentrations (pg·mL^–1^) were calculated from a standard curve ranging from 0 to 6000 pg·mL^–1^.

#### 2.4.3 Statistical Analysis

All statistical evaluations were carried out using GraphPad Prism software, version 8.0 (GraphPad Software Inc., San Diego, CA, USA). Data were analyzed by one-way analysis of variance (ANOVA), and differences among the groups were determined using Tukey’s multiple comparison test, adopting a significance level of *p* < 0.05. Results represent the mean values obtained from three independent experiments for the anti-inflammatory and mucoadhesive assays, and from four replicates for the mechanical testing.

## 3 Results and Discussion

### 3.1 Characterization of Membranes

#### 3.1.1 Scanning Electron Microscope (SEM)

Figure 1 shows the micrographs of the surfaces of CH and CH/ArEO membranes examined by scanning electron microscope.

**Figure 1:**
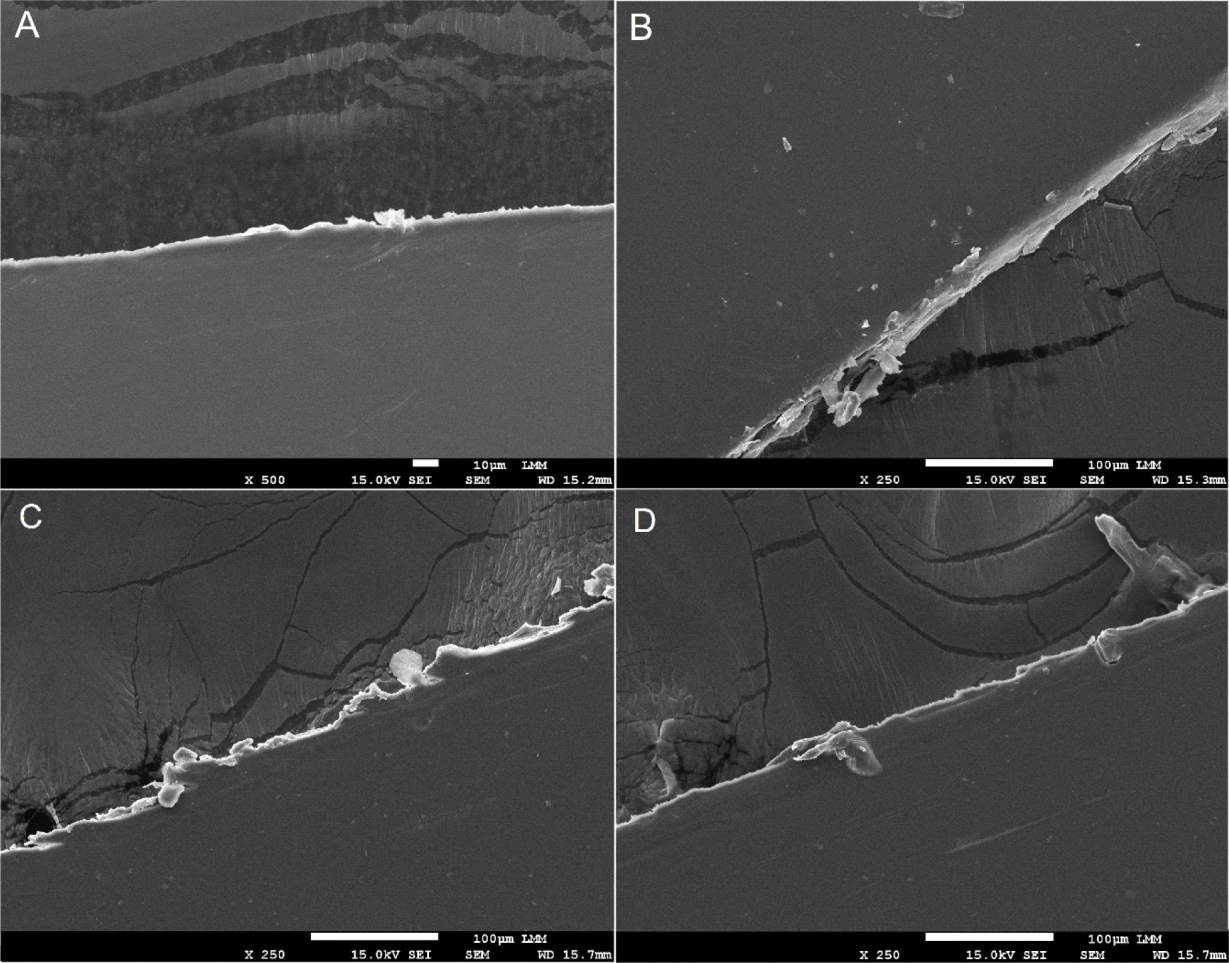
Scanning electron microscope (SEM) micrographs of the surfaces of CH and CH/ArEO membranes: CH (A), scale bar = 10 μm; CH/ArEO 0.5%, scale bar = 100 μm (B); CH/ArEO 2.5%, scale bar = 100 μm (C); CH/ArEO 5.0% (D), scale bar = 100 μm.

The surface analysis of the membranes shows a flat, dense, and compact surface. The denser structure may result from a homogeneous distribution of ArEO and the drying process. The concentration of ArEO did not influence the morphology of the samples. No surface pores were observed due to the potential volatilization of ArEO in the chitosan matrix. The dense surface of the membrane serves as an effective barrier, blocking microbes from entering and multiplying in the wound.^34^

Similar results were observed in another study when at 1.5% lemongrass EO (*Cymbopogon citratus*) rich in neral or β-citral, and geranial or α-citral was added to the chitosan film, presenting a uniform morphology.^35^ Similarly, the incorporation of rue essential oil (*Ruta graveolens*), which contains 2-nonanone and 2-undecanone, into chitosan films has been reported to produce a smooth and uniform surface with only minor fissures.^9^ In contrast, carboxymethyl chitosan and gelatin films containing EO of *Litsea cubeba* rich in α-citral, β-citral, d-limonene, α-pinene, β-pinene, β-phellandrene, linalool, citronellal, and camphene, exhibited a rougher surface, which has been attributed to structural disturbances induced by the active compounds.^14^

#### 3.1.2 Thermogravimetric Analysis (TGA)

The mass loss and its derivative for the membranes examined are illustrated in Figure 2A,B, respectively. Table 1 presents the peak decomposition temperature (*T*_dmax_), the initial degradation temperatures (*T*_onset_), the mass loss values, and the residue percentages.

**Figure 2:**
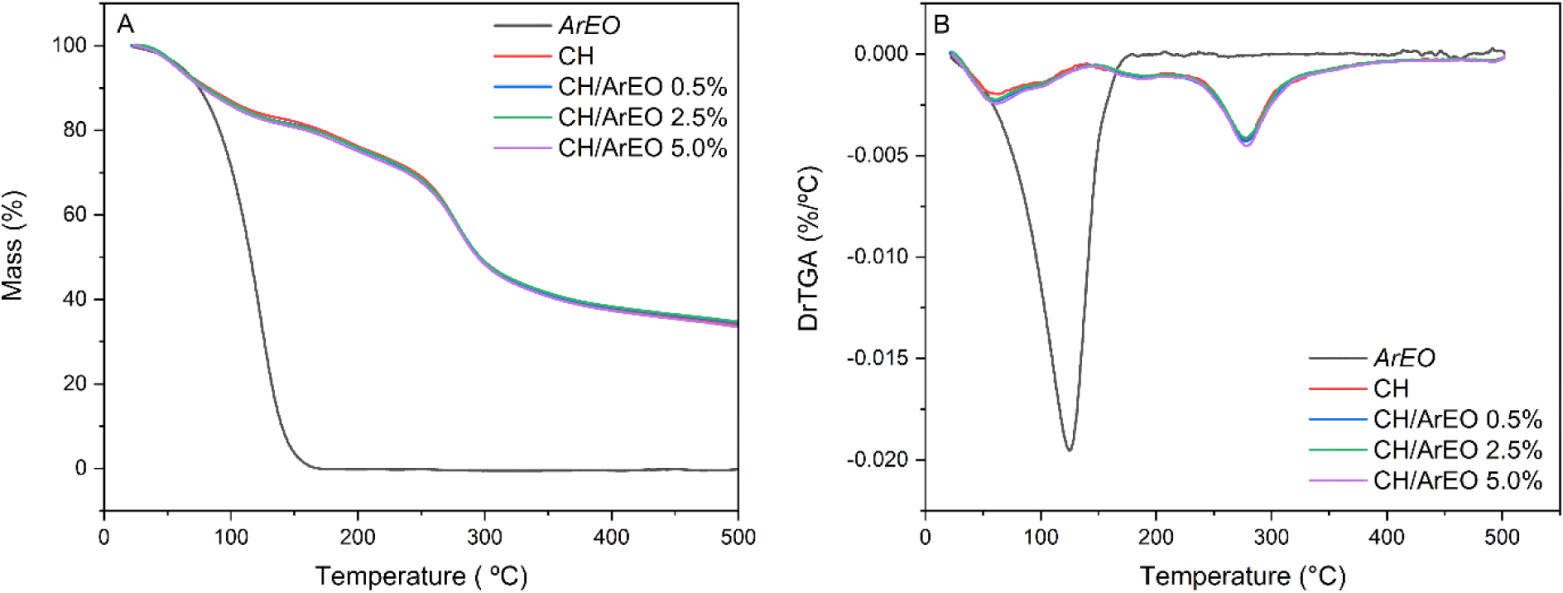
TGA (A) and DrTGA (B) from ArEO, CH, CH/ArEO 0.5%, CH/ArEO 2.5%, and CH/ArEO 5.0% samples were performed from 25 to 500 °C at a heating rate of 10 °C/min in a nitrogen atmosphere.

**Table 1:** Thermogravimetric (TGA) and Derivative Thermogravimetric (DTG) Parameters of the Membranes and ArEO, Including Degradation Onset Temperature (*T*_onset_), Maximum Degradation Temperature (*T*_dmax_), Weight Loss (%), and Residual Mass (%)

	***T*_onset_** (°C)			***T*_dmax_** (°C)				
**Samples Code**	**1° Stage**	**2° Stage**	**3° Stage**	**1° Stage**	**2° Stage**	**3° Stage**	**Total Weight Loss (%)**	**Residue (%)**
ArEO	25.01			174.35			99.69	0.31
CH	25.93	141.90	235.95	140.30	234.08	501.82	66.19	33.81
CH/ArEO 0.5%	21.84	150.03	238.44	150.03	238.44	500.98	65.73	34.27
CH/ArEO 2.5%	24.40	144.08	228.96	144.08	228.96	499.93	65.60	34.40
CH/ArEO 5.0%	24.17	143.92	231.87	143.92	231.87	499.72	66.52	33.48

The thermogram obtained by the ArEO TGA highlights the sample’s decomposition in a single stage. By analyzing their data, it can be observed that the largest fraction of linalool (99.69%) decomposes and volatilizes up to 174.35 °C.^36^ The decomposition temperature of ArEO was above the processing temperature of the materials.^37^

The CH membranes with and without ArEO underwent degradation in three stages. The effective incorporation of ArEO into the chitosan membrane is likely associated with intermolecular interactions between the functional groups of the oil constituents and the amino (−NH_2_) and hydroxyl (−OH) groups of the polymer matrix, which promote hydrogen bonding,^38^ resulting in an excellent protective effect and prevents the separate decomposition of ArEO. This indicates that the ArEO remains stable within the chitosan matrix and does not volatilize or degrade easily at typical storage conditions, enhancing the membrane’s long-term performance.

The first stage involves a mass loss of approximately 18.33%, occurring between 21.84 and 25.93 °C and 140.30–150.03 °C, primarily due to the loss of water, acetic acid, and some volatile compounds, peaking at 60 °C.^39,40^ The second stage, with about 10.23% mass loss, occurs from 141.90 to 150.03 °C to 228.96–238.44 °C, associated with water evaporation from chitosan and the volatilization of essential oil components, peaking at 180.32 °C.^41^ The main degradation stage occurs between 228.96 and 238.44 °C to 499.72–501.82 °C, with a mass loss of roughly 36.66%, attributed to the loss of saccharide units, dehydration, depolymerization, and degradation of stable essential oil components, peaking at 277.81 °C.^9^

Comparable three-phase breakdown processes have been noted in other films. Cellulose nanofibrils reinforced chitosan-gelatin based hydrogel and oregano essential oil (thymol, γ-terpinene, isopropyl *o*-cresyl sulfide, (*E*)-caryophyllene, linalool, and β-myrcene) for diabetic wound healing, also presented three-step degradation, with the second step (150 to 270 °C) and nearly 60% weight loss result from the decomposition of essential oil and polymer decarboxylation.^42^ Carboxymethyl chitosan and gelatin films containing EO of *Litsea cubeba* presented with the main degradation stage between 153.68 and 542.28 °C and a mass loss of 72% attributed to the breakdown of amino and hydroxymethyl functionalities within the polymer matrix, along with the loss of high-boiling volatile constituents from the essential oil, such as d-limonene, citral, linalool, and citronellal.^14^

According to the TGA thermograms of the CH membranes, the mass loss due to evaporation showed a small difference due to the incorporation of ArEO. The mass loss at 100 °C about the ArEO concentration was 13.73% for 0.5%, 13.64% for 2.5%, and 14.18% for 5.0%, against 12.91% for the oil-free CH.

The incorporation of ArEO up to 5.0% did not alter the thermal stability of the membranes, as the onset of degradation and the main mass-loss events remained essentially unchanged compared to neat chitosan. This pattern aligns with earlier research indicating that incorporating essential oils, even at higher concentrations, usually produces only minor variations in *T*_onset_ and *T*_max_ values.^43,44^ It should be noted, however, that increasing the concentration of EO beyond 5% tends to compromise the physic mechanical performance of chitosan membranes due to phase separation and plasticizing effects, resulting in reduced tensile strength, higher porosity, although elongation at break may increase. CS/PVA films containing 10% clove EO exhibited discontinuities in the polymer matrix and greater mass loss compared with 1%, leading to reduced heat resistance and impaired structural stability.^45^

#### 3.1.3 Fourier Transform Infrared Spectroscopy (FTIR)

Figure 3 shows the Fourier transform infrared (FTIR) spectra of the ArEO incorporated CH membranes for different ArEO concentrations in the wavelength range of 4000–500 cm^–1^.

**Figure 3:**
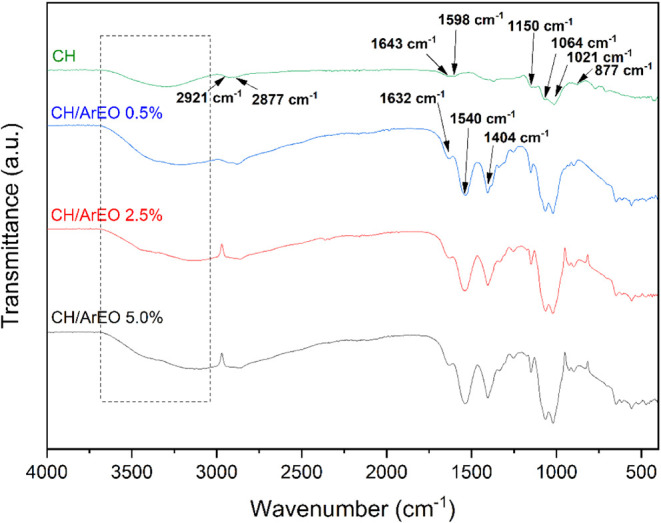
Fourier transform infrared spectroscopy (FTIR) spectra of the CH and CH/ArEO membranes.

The FTIR spectrum of the CH membrane exhibits a broad absorption band in the range of 3676–3002 cm^–1^, attributed to O–H stretching vibrations, which overlap with N–H vibrations, indicating the presence of intermolecular hydrogen bonding interactions.^46^ The bands observed at 2921 and 2877 cm^–1^ correspond to the asymmetric and symmetric stretching modes of C–H bonds, respectively.^35^ Characteristic signals of chitosan were detected between 1643 and 1598 cm^–1^, associated with the C=O stretching of the amide I group and the N–H bending of the amide II group. The band at 1404 cm^–1^ is assigned to the HN–CO stretching vibration of the amide III group;^45,47^ while the symmetric bending of methyl (CH_3_) groups is observed at 1369 cm^–1^.^48^ The peak at 1150 cm^–1^ corresponds to the asymmetric stretching of the C–O–C glycosidic bond.^9,35^ Furthermore, C–O stretching vibrations appear at 1064 and 1021 cm^–1^, coupled with adjacent C–C bond deformation. The signal at 877 cm^–1^ is attributed to C–O–C stretching.^49^

The inclusion of ArEO led to minor variations in the spectra when compared to CH control. The bands described referred to the amide group, and those of the C–H stretching vibration increased their intensity, which was also observed in another study.^9^ Furthermore, the peak at 1632 cm^–1^ is associated with C=C stretching in components such as linalool, abundant in the ArEO.^34,50^ The chitosan/ArEO spectrum showed stronger and elongated bands.

A distinct absorption band at 1252 cm^–1^, attributed to the axial stretching of C–O bonds, was identified in the spectra of membranes containing the essential oil. This peak is likely associated with the presence of linalool, a major component of ArEO.^51–53^ Apart from this, no significant emergence of new bands or noticeable shifts in existing peaks were observed in the FTIR spectrum of the ArEO-loaded CH membrane. These results suggest the occurrence of intermolecular hydrogen bonding interactions between chitosan and ArEO,^42,54^ which may contribute to reducing the premature release of the essential oil and thus allow the maintenance of the biological activities of the incorporated membranes during wound healing.

#### 3.1.4 Mechanical Properties

For modern wound dressings, mechanical properties are essential to provide appropriate physical support for cell proliferation and growth.^55^ For this purpose, the mechanical properties, including breaking force, elongation, breaking stress, deformation, and Young’s modulus for CH and CH/ArEO membranes were determined and reported in Table 2. Table 2 also shows the mucoadhesive capacity of membranes.

**Table 2:** Breaking Force, Elongation, Breaking Stress, Deformation, Young’s Modulus, and Mucoadhesive of ArEO Incorporated CH Membranes^a^

**Sample Code**	**Breaking force (N)**	**Elongation (mm)**	**Breaking stress (MPa)**	**Deformation (%)**	**Young’s Modulus (MPa)**	**Mucoadhesion (N)**
CH	40.21 ± 3.39	2.07 ± 0.22^a^	1.34 ± 0.11	6.91 ± 0.74^a^	48.00 ± 6.26	0.161 ± 0.029
CH/ArEO 0.5%	41.20 ± 5.78	4.30 ± 0.60^b^	1.37 ± 0.19	14.34 ± 2.01^b^	46.25 ± 5.74	0.118 ± 0.024
CH/ArEO 2.5%	40.66 ± 2.60	3.36 ± 1.00^a,b^	1.36 ± 0.09	11.20 ± 3.35^c,b^	41.84 ± 7.19	0.145 ± 0.035
CH/ArEO 5.0%	40.05 ± 5.94	3.17 ± 0.53^a,b^	1.34 ± 0.20	10.57 ± 1.78^d,e^	43.68 ± 7.04	0.135 ± 0.008

a ^a,b,c,d,e^Different letters in the same column indicate a significant difference by Tukey’s test (*p* ≤ 0.05).

The elongation at break test measures the ability of the film to withstand elongation and deformation prior to rupture. The results suggest that incorporating ArEO tends to increase the elongation at break. At the lowest concentration of ArEO in the formulation, the variation in elongation at break is greater (control: 6.91%; CH/ArEO 0.5%: 14.34%). However, a significant increase (*p* ≤ 0.05) in elongation at break of up to approximately 34% is apparent at the highest concentration of ArEO in the formulation (10,57%). This change suggests that ArEO could improve the ductility and flexibility of the formulated membrane, allowing it to stretch further before breaking under stress.^56^ The functional groups of ArEO interacted with the CH chains and weakened the intramolecular forces, resulting in the increased mobility and flexibility of the chitosan membrane.^57^ A similar phenomenon was observed in the study of nanoemulgel chitosan, polyvinylpyrrolidone, and gelatin incorporated with oregano EO containing thymol, γ-terpinene, isopropyl *o*-cresyl sulfide, (*E*)-caryophyllene, linalool, and β-myrcene.^42^ The chitosan/gelatin/poly­(vinyl alcohol) film incorporated with *Thymus pubescens* EO rich in carvacrol, linalool, and thymol also showed increased deformation (1.4% to 19.26%).^56^

Young’s modulus quantifies the stiffness of a material, indicating its resistance to deformation when subjected to an external force. This parameter is particularly relevant for wound dressing applications, as it affects the material’s capacity to preserve structural stability while remaining sufficiently flexible to adapt to the contours of the wound site.^58^ Like the rupture stress, Young’s modulus did not significantly differ by incorporating ArEO (*p* < 0.05), denser structures tend to exhibit greater rigidity and consequently higher modulus values compared to more porous matrices.^55^ A similar trend was reported in bioactive chitosan films with lemongrass (*Cymbopogon citratus*) rich in β-citral and α-citral.^35^ The CH and CH/ArEO membrane samples demonstrated Young’s modulus of elasticity ranging from 41.84 to 48.00 MPa, confirming the resistance of the dressings to deformation under stress. The high value of Young’s modulus may attribute a greater resistance of the material to elastic deformation, and the values found include the values of the elastic modulus of the skin (0.02 to 57 MPa).^59,60^ Several variables, including the degree of chitosan deacetylation, the pH of the precursor solution, the presence of plasticizing agents, and specific parameters of the synthesis process, influence the mechanical behavior of membranes and films.^3^ The breaking stress of CH and CH/ArEO ranging from 1.34 to 1.37 MPa, which can conform to the contours of the wound site and adapt to the movements of the surrounding skin without causing discomfort or detachment.

The area under the force–distance curve, which represents the energy required to detach the probe from the membrane surface, is defined as the mucoadhesive work. Mucoadhesive membranes are specifically engineered to maintain prolonged contact with biological tissues, enabling a more controlled and sustained release of active compounds.^61^ According to the data presented in Table 2, the incorporation of ArEO did not lead to a statistically significant change in the mucoadhesive performance of the chitosan-based membranes. The data demonstrate a mucoadhesive characteristic linked to the inclusion of chitosan in the formulations, as it enhances the duration that the membranes remain on the mucin discs.^62^

Chitosan’s mucoadhesive behavior is primarily attributed to electrostatic interactions between its positively charged amino groups and the negatively charged sites on mucin, resulting in the formation of an interfacial electrostatic double layer that promotes adhesion.^63^ Additionally, chitosan can modulate mucosal permeability by disrupting tight junctions through calcium ion chelation, which occurs via interactions with calcium channels in the mucosal tissue.^64^ The mucoadhesive property of chitosan is an essential feature for enhancing the skin permeation of EOs.^65^ In previous studies, chitosan-based mucoadhesive films not only demonstrated sufficient tissue adhesion but also facilitated increased curcumin penetration into the oral mucosa, supporting their potential in localized therapies for oral cancer.^66^

The results obtained highlight the benefits of the developed formulation over conventional treatments, which often require frequent reapplication.^66^ This characteristic makes it particularly suitable for use in wound dressings, as it allows for direct application to the injury site without inducing further irritation or tissue damage.^3,30^

### 3.2 Biological Tests

#### 3.2.1 Cell Viability Analysis

Effects of different concentrations of ArEO alone and incorporated into chitosan (CH) on the viability of RAW 264.7 macrophages are shown in Figure 4A,B.

**Figure 4:**
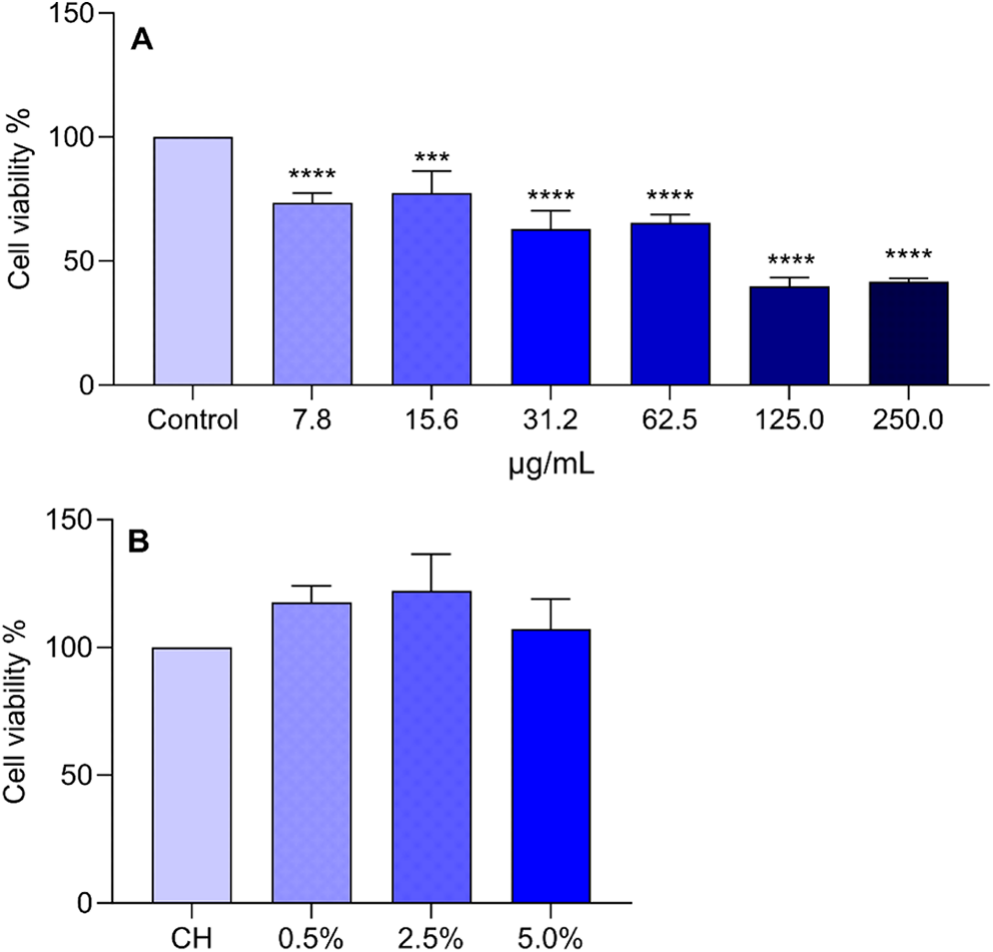
(A) Effects of different concentrations of *Aniba rosaeodora* Ducke oil and (B) incorporated into chitosan (CH) on the cell viability of RAW 264.7 macrophages after 24 h of treatment. Data are represented as mean ± SD in triplicate from three independent experiments. ****: *p* < 0.0001; ***: *p* < 0.001 indicates a significant difference compared to the control (0.01% DMSO); *: *p* < 0.05 indicates a significant difference compared to the CH, analyzed by one-way ANOVA followed by Tukey’s test.

*Aniba rosaeodora* Ducke essential oil (ArEO) exhibited a cytotoxic effect at all tested concentrations compared to the control. Therefore, concentrations below 7.81 μg/mL were selected for subsequent assays (Figure 4A).

The incorporation of ArEO into CH did not result in any significant alteration in cell viability across all tested concentrations when compared to CH alone (Figure 4B).

#### 3.2.2 In Vitro Anti-Inflammatory Activity

##### 3.2.2.1 Nitrite Dosage

The nitrite assay analysis of ArEO and its incorporation into chitosan is shown in Figure 5A,B.

**Figure 5:**
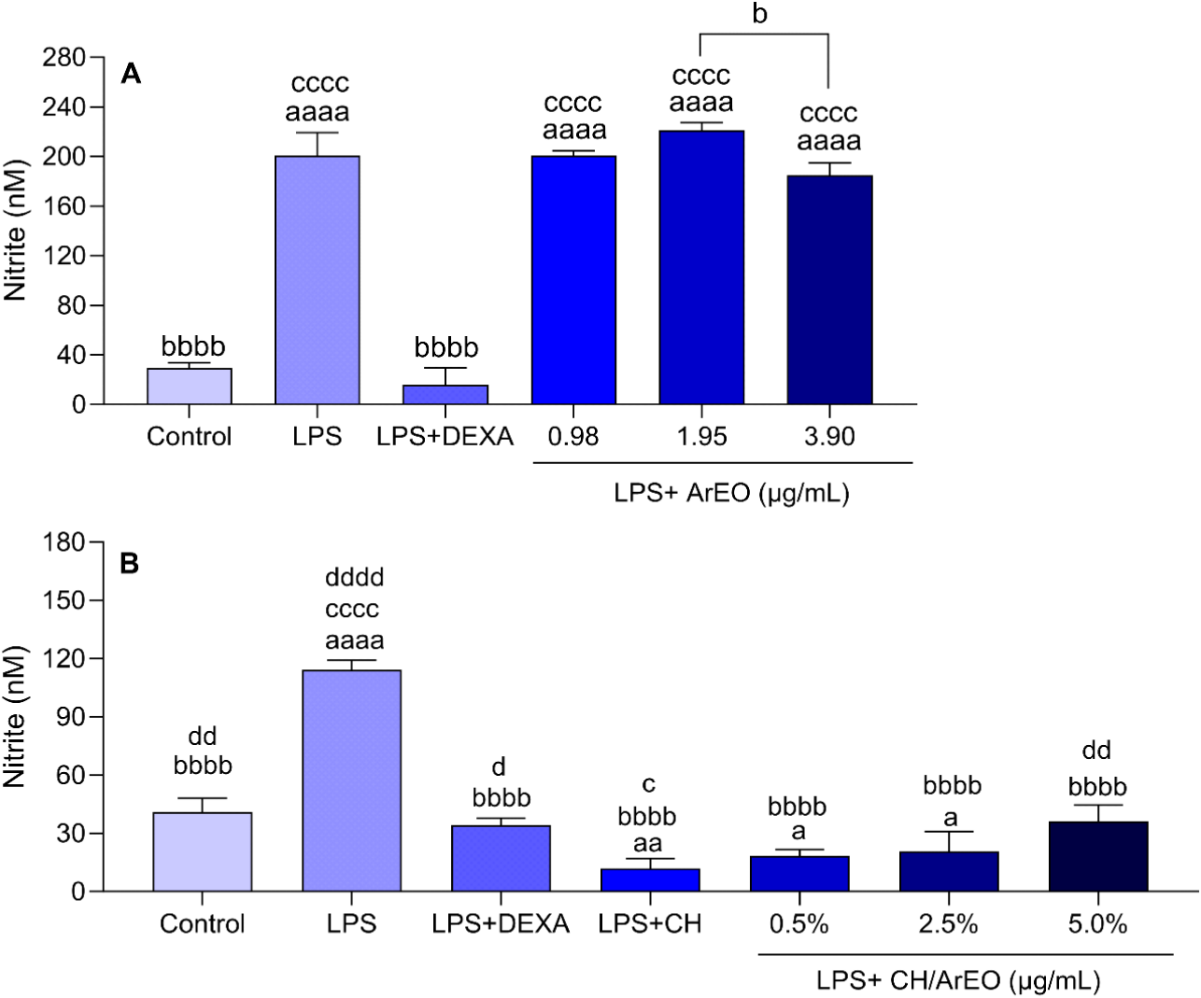
(A) Effect of *Aniba rosaeodora* essential oil (ArEO) and (B) incorporated in chitosan (CH/ArEO) on nitrite production (nM) in RAW 264.7 macrophages stimulated by LPS (0.5 μg/mL). Data are represented by mean ± SD of three independent experiments in triplicate. aaaa: *p* < 0.0001, aa: *p* < 0.01, a: *p* < 0.05 indicates significant differences compared to the control (0.01% DMSO). bbbb: *p* < 0.0001, b: *p* < 0.05 indicates significant differences compared to the LPS group and between groups treated with ArEO previously stimulated by LPS. cccc: *p* < 0.0001; c: *p* < 0.05 indicates significant differences compared to the group stimulated by LPS and subsequently treated with dexamethasone (DEXA, 10 μM). dddd: *p* < 0.0001, dd: *p* < 0.01, d: *p* < 0.05 indicates significant differences compared to the group stimulated by LPS and subsequently treated with chitosan (LPS + CH), analyzed by one-way ANOVA followed by Tukey’s test.

Nitrite level analysis confirmed the successful induction of the inflammatory model in RAW 264.7 macrophages, as lipopolysaccharide (LPS)-stimulated cells showed a significant increase in nitrite production compared to the negative control. Dexamethasone, used as a positive control due to its recognized anti-inflammatory activity, significantly reduced nitrite levels compared to the LPS-treated group (Figure 5A,B).

Treatment with ArEO did not significantly differ in nitrite concentrations at any tested concentrations compared to the LPS group. Moreover, all groups treated with ArEO exhibited significantly elevated nitrite levels compared to both the control and LPS + DEXA groups (Figure 5A), suggesting that, at low concentrations, ArEO did not demonstrate an anti-inflammatory effect. Previous studies involving cells stimulated by *Trypanosoma cruzi* showed that treatment with *Aniba rosaeodora* essential oil, which contains 93.60% linalool, did not lead to a significant reduction in nitrite production.^67^ Similarly, in vitro assays using macrophages exposed to linalool at concentrations of 250 or 350 μg/mL—either prior to or following interaction with *Leishmania infantum*—revealed no notable changes in nitric oxide levels.^68^ However, studies with RAW264.7 macrophage cells treated with LPS demonstrated significant inhibition of nitric oxide production compared to the LPS control group, at all linalool concentrations (10, 50, and 100 μM).^69^

The incorporation of ArEO into the chitosan matrix led to a statistically significant reduction in nitrite concentrations at all evaluated concentrations (0.5%, 2.5%, and 5%) when compared to the LPS-stimulated group (*p* < 0.0001). Additionally, a marked decrease in nitrite levels was detected in the LPS + CH group compared to both the LPS (*p* < 0.0001) and LPS + DEXA (*p* < 0.05) groups, suggesting that the observed anti-inflammatory activity may be attributed to the intrinsic biological properties of the chitosan, potentially surpassing the effect of dexamethasone. Furthermore, at the highest concentration tested (5%), nitrite levels were significantly elevated compared to those observed in the LPS + CH group (*p* < 0.01), indicating that increasing the concentration of ArEO incorporated into CH may attenuate its anti-inflammatory effect (Figure 5B).

##### 3.2.2.2 Measurement of Tumor Necrosis Factor-Alpha (TNF-α) through ELISA

The TNF-α-level assay analysis of ArEO and its incorporation into chitosan is shown in Figure 6A,B.

**Figure 6:**
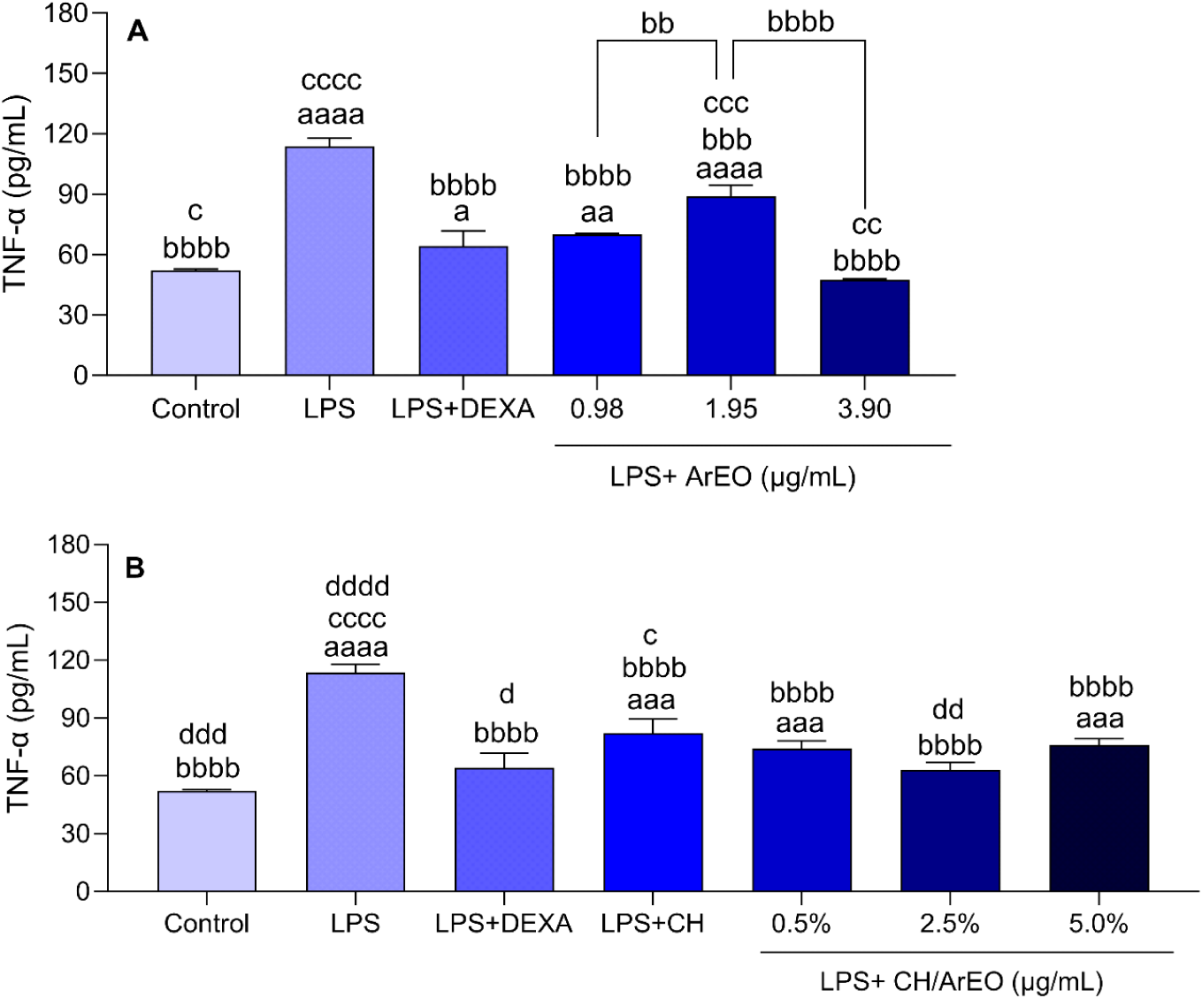
(A) Effect of *Aniba rosaeodora* essential oil (ArEO) and (B) incorporated in chitosan (CH/ArEO) on TNF-α levels (pg/mL) in RAW 264.7 macrophages stimulated by LPS (0.5 μg/mL). Data represented by mean ± SD of three independent experiments in triplicate. aaaa: *p* < 0.0001, aaa: *p* < 0.001, aa: *p* < 0.01, a: *p* < 0.05 indicates significant differences compared to the control (0.01% DMSO). bbbb: *p* < 0.0001, bbb: *p* < 0.001, bb: *p* < 0.01 indicates significant differences compared to the LPS group and between groups treated with ArEO previously stimulated by LPS. cccc: *p* < 0.0001, ccc: *p* < 0.001; cc: *p* < 0.01, c: *p* < 0.05 indicates significant differences compared to the group stimulated by LPS and subsequently treated with dexamethasone (DEXA, 10 μM). dddd: *p* < 0.0001, ddd: *p* < 0.001, dd: *p* < 0.01 indicates significant differences compared to the group stimulated by LPS and subsequently treated with chitosan (LPS + CH), analyzed by one-way ANOVA followed by Tukey’s test.

TNF-α levels were significantly elevated in RAW 264.7 macrophages following LPS stimulation relative to the negative control group (*p* < 0.0001), thereby validating the induction of the inflammatory response. As expected due to its anti-inflammatory properties, dexamethasone treatment (LPS + DEXA group) led to a significant reduction in TNF-α production compared to the LPS group (*p* < 0.01) (Figure 6A,B).

At all tested concentrations of ArEO, a significant decrease in TNF-α levels was observed compared to the LPS group (*p* < 0.05), with reductions of 38.4% at 0.98 μg/mL, 21.6% at 1.95 μg/mL, and 58.1% at 3.9 μg/mL. However, TNF-α levels at 1.95 μg/mL were significantly higher than those observed at 0.98 μg/mL, 3.9 μg/mL, and in the LPS + DEXA group. Furthermore, treatment with the highest concentration of ArEO (3.9 μg/mL) significantly reduced TNF-α levels compared to the LPS + DEXA group (Figure 6A). In another investigation, linalool—the primary component of *Aniba rosaeodora* essential oil employed in this study—exhibited a notable anti-inflammatory effect by significantly reducing the production of the pro-inflammatory cytokine TNF-α in LPS-stimulated RAW 264.7 macrophages in vitro.^70^

Similarly, treatment with CH/ArEO reduced TNF-α levels at all tested concentrations, with decreases of 34.7%, 44.4%, and 33% compared to the LPS group. Additionally, the LPS + CH group exhibited lower TNF-α levels than the LPS group, but higher levels than those observed in the LPS + DEXA group, confirming the anti-inflammatory potential of the polymer alone. However, a further decrease in TNF-α levels relative to the LPS + CH group was observed only with the formulation containing 2.5% ArEO incorporated into the chitosan matrix, suggesting that, depending on the concentration, CH alone may exert an effect comparable to or even greater than that of the CH/ArEO formulation (Figure 6B).

Lipopolysaccharide (LPS) activates RAW 264.7 macrophages through binding to the CD14/TLR4/MD2 receptor complex located on the cell surface, initiating signaling pathways that stimulate the synthesis of numerous inflammatory mediators, including IL-1α/β, IL-6, IL-12, COX-2 (cyclooxygenase-2), ROS/RNS, and TNF-α.^71,72^ In this study, the effects of LPS stimulation were evaluated by quantifying TNF-α expression and nitrite (NO_2_^–^), a stable product of nitric oxide (NO) metabolism. TNF-α acts through TNF receptor 1, activating NF-κB signaling and promoting its nuclear translocation, which in turn induces the transcription of pro-inflammatory genes encoding cytokines, chemokines, and enzymes, including COX-2, PLA2 (phospholipase A2), and iNOS or NOS2.^73^

In macrophages and microglial cells, NO is synthesized by iNOS by converting l-arginine to NO and citrulline. However, due to its short half-life (∼30 s), NO is rapidly metabolized, often reacting with superoxide (O_2_^–^) to form peroxynitrite, a highly cytotoxic species associated with cell death.^73^ In the present findings, treatment with ArEO significantly reduced TNF-α levels without altering nitrite concentrations. This dissociation suggests that the compound may selectively modulate upstream signaling pathways, particularly those involved in cytokine production, such as TNF-α, while not directly affecting iNOS expression or NO production, which is reflected in the stable nitrite levels.

Moreover, the findings of this study indicate that treatment with chitosan and *Aniba rosaeodora* essential oil (CH/ArEO) reduces nitrite and TNF-α levels. These results emphasize the multifunctional nature of chitosan, which exhibits antibacterial, hemostatic, antioxidant, and anti-inflammatory activities. This versatility has made chitosan highly sought after for various biomedical applications.^74^

Terpenoids work by inhibiting enzymes and modulating the immune system to reduce inflammation.^75,76^ Some research indicates that the antioxidant capabilities of bioactive compounds can effectively reduce inflammation by neutralizing oxidative damage.^77^ Linalool, a compound found in *Aniba rosaeodora* essential oil, has powerful antioxidant properties, which help neutralize free radicals and protect cells from oxidative stress,^78^ which may protect against disease by reducing the production of pro-inflammatory cytokines and other inflammatory markers.^79^ Furthermore, linalool can reduce inflammation, alleviate heightened pain sensitivity, and provide pain relief.^19,80–85^ Another study analyzing reverse transcription-polymerase chain demonstrated a notable downregulation of pro-inflammatory markers such as IL-1β, IRAK, NF-κB, TNF-α, and IL-17, alongside an upregulation of the anti-inflammatory cytokine IL-10, in linalool-treated rats compared to controls.^86^ Therefore, combining chitosan with linalool-rich *Aniba rosaeodora* essential oil may further aid in reducing inflammation and promoting wound healing.

Compared to conventional anti-inflammatory therapies, such as corticosteroids and NSAIDs, which effectively reduce inflammation but are often associated with cytotoxicity, delayed tissue regeneration, and systemic side effects,^87^ the CH/ArEO membrane offers a safer and more localized alternative. The system provides sustained delivery of natural bioactive compounds directly to the wound site, resulting in significant reductions in nitrite and TNF-α levels without compromising cell viability. This localized and biomaterial-based approach not only achieves anti-inflammatory efficacy comparable to that of conventional agents but also supports tissue repair through the combined antioxidant and bioadhesive properties of chitosan and the bioactive terpenoids present in ArEO. Therefore, the CH/ArEO membrane represents a promising natural strategy that integrates inflammation control with enhanced wound-healing potential.

## 4 Conclusions

Developing chitosan membranes combined with *Aniba rosaeodora* EO for wound healing represents a promising advancement. These membranes are characterized by their flexibility, homogeneity, and continuous structure, features that favor their interaction with irritated wound sites. Their mucoadhesive properties ensure strong tissue adhesion without significant differences observed when added EO. *Aniba rosaeodora* oil significantly reduces nitrite levels in an in vitro inflammation model, especially when incorporated into chitosan. Furthermore, TNF-α levels in LPS-stimulated macrophages decrease significantly following membrane application, containing 0.5%, 2.5% and 5% of the essential oil. These results underscore the potential of utilizing *Aniba rosaeodora* EO in conjunction with chitosan to create advanced dressings, effectively addressing the limitations of traditional wound dressings. It is important to highlight that more tests related to the migration of compounds and their interaction mechanisms with biological tissues, as well as in vivo tests and applied to the human body, are necessary to maximize the membrane’s ability to be used for the proposed application.

## Supplementary Material



## Notes

Characterization of the viscosity of the samples; macroscopic image of the synthesized membrane (PDF)
