# Supplementary material for: Anti-Inflammatory Effect of Aniba rosaeodora Essential Oil-Loaded Chitosan Membrane
Source: ACS Omega. 2026 Feb 18;11(8):13094–106. doi: 10.1021/acsomega.5c06864 (PMC12961530; doi:10.1021/acsomega.5c06864)

Anti-inflammatory effect of *Aniba rosaeodora* essential oil-loaded chitosan membrane

*Bruna Michele A. de B. Buriti [1], Caio Augusto de A. Canelas [2], Venâncio A. Amaral [3], Laine Celestino Pinto [4], Renata C. Silva [5], William N. Setzer [6], Tais Gratieri [3], Pablo Luis B. Figueiredo [2], Marcele F. Passos [2,7] and Joyce Kelly R. da Silva [1,7] **

[1] Instituto de Ciências Exatas e Naturais, Programa de Pós-Graduação em Química, Universidade Federal do Pará, Belém 66075-110, PA, Brazil; bruna.brito@icen.ufpa.br
[2] Programa de Pós-Graduação em Ciências Farmacêuticas, Universidade Federal do Pará, Belém 66079-420, PA, Brazil; caio.a.canelas@gmail.com (C.A.A.C.); pablo.figueiredo@uepa.br (P.L.B.F.); cellepassos@ufpa.br (M.F.P)
[3] Laboratório de Alimentos, Medicamentos e Cosméticos (LTMAC), Universidade de Brasília, Brasília 70910-900, DF, Brasil; venancio.aa@gmail.com (V.A.A.); tgratieri@unb.br (T.G.)
[4] Laboratório de Neuropatologia Experimental, Hospital Universitário João de Barros Barreto, Universidade Federal do Pará, 66073-000, Belém, PA, Brazil; lainecelestino@hotmail.com
[5] Programa de Pós-Graduação em Biologia Parasitária na Amazônia, Universidade Estadual do Pará, Belém 66095-662, PA, Brazil; renatacsterapeuta@gmail.com
[6] Aromatic Plant Research Center, 230 N 1200E, Suite 100, Lehi, UT 84043, USA; setzerw@uah.edu
[7] Programa de Pós-Graduação em Biotecnologia, Universidade Federal do Pará, Belém 66075-110, PA, Brazil.
[*] Correspondence: joycekellys@ufpa.br

Supplementary Material

Characterization of the viscosity of the samples

The viscosity of the samples was measured using a rotational viscometer (MVD-5, Marte Científica) equipped with a SP0 spindle, operated at a constant speed of 12 rpm. Measurements were performed at room temperature (25 ± 1 °C).

Table S1. The results of the viscosity of the samples.

Samples	Viscosity (mPa·s)
CH	24.2
CH/ArEO 0.5%	24.2
CH/ArEO 2.5%	24.2
CH/ArEO 5.0%	24.2

Figure S1 – Macroscopic image of synthesized membrane (thickness: 0.24±0.016 mm).

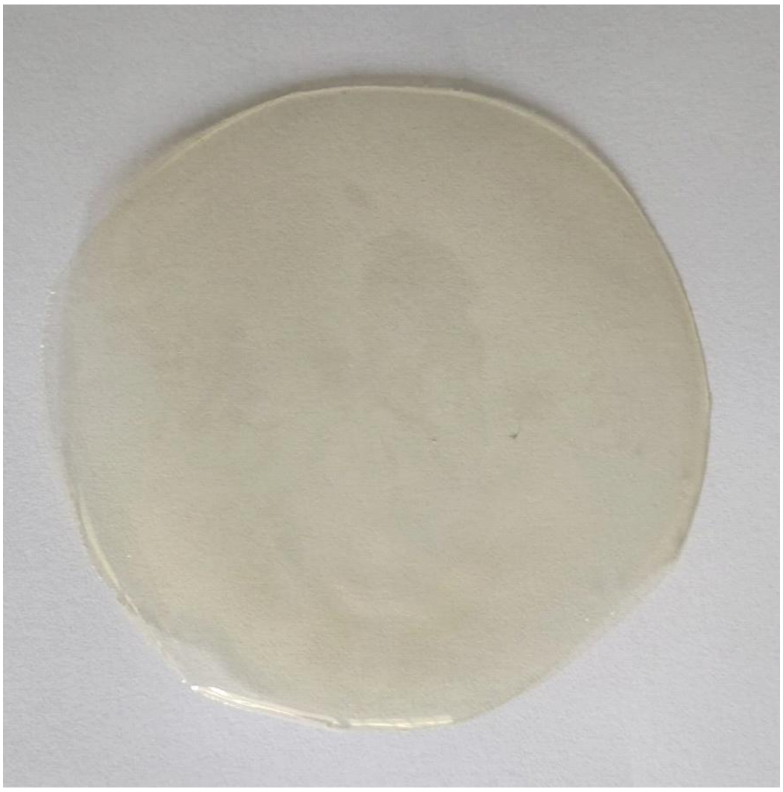

Supplement: Supplementary file 1 [file ao5c06864_si_001.pdf]
